# Supplementary material for: Baseline Imaging Derived Predictive Factors of Response Following [177Lu]Lu-PSMA-617 Therapy in Salvage Metastatic Castration-Resistant Prostate Cancer: A Lesion- and Patient-Based Analysis
Source: Biomedicines. 2022 Jul 1;10(7):1575. doi: 10.3390/biomedicines10071575 (PMC9313142; doi:10.3390/biomedicines10071575)
Supplement: Supplementary file 1 [file biomedicines-10-01575-s001.zip › biomedicines-1781425-supplementary.pdf]

PSMA-PET reponse - mixed model analysis

S.C. Ebbers

28 June 2022

Import the data

Import the data in long format, labeling the tumors within the patient.

```
source("data_import.r")
```

Visualize the data

Plot tables, histogram and a spaghettiplot of every tumor with it's SUV values over time.

```
table(data.long$response.cat)

hist(filter(data.long, tumor_hotness == "1" | tumor_hotness ==
    "2")$S1Peak.orig)

hist(filter(data.long, tumor_hotness == "3" | tumor_hotness ==
    "4")$S1Peak.orig)

ggplot(data = data.ultralong, aes(x = Timepoint, y = SUV)) +
    geom_line(aes(group = interaction(ID, metastasis_type, tumor_hotness,
        SUVtype), color = metastasis_type)) + scale_y_log10()
```

Transform (log)

If needed (probably so) the SUV-values can be log-transformed in order to be able to fit a model.

```
data.long <- data.long %>%
    mutate(S1Max = log(S1Max.orig), S1Peak = log(S1Peak.orig))
```

Create additional datasets

Prepare multiple datasets to test on subgroups if needed. Also, tumors without a value are removed, as a random effects model will fail otherwise.

```
dose_SUVPEAK <- data.long %>%
    filter(!is.na(S1Peak) & !is.na(response))
```

```
dose_SUVMAX <- data.long %>%
    filter(!is.na(S1Max) & !is.na(response))

dose_SUVPEAK.high <- dose_SUVPEAK %>%
    filter(S1Peak.orig > 14.87)

dose_SUVPEAK.low <- dose_SUVPEAK %>%
    filter(S1Peak.orig <= 14.87)

dose_SUVMAX.high <- dose_SUVMAX %>%
    filter(S1Max.orig > 19.08)

dose_SUVMAX.low <- dose_SUVMAX %>%
    filter(S1Max.orig <= 19.08)
```

Models fitted with ML

To test models, the lme-function is used to fit a model with and without the response as dependent variable, and with and without a random intercept. Using the anova-functions, models can be compared to each other, where models with a lower AIC are better. This can be repeated for different subgroups or different measurement (i.e. SUV-max instead of SUV-peak)

```
fit0 <- lme(fixed = S1Peak ~ 1, random = ~1 | ID, data = dose_SUVPEAK,
    method = "ML", na.action = na.omit)
fit1 <- lme(fixed = S1Peak ~ response, random = ~1 | ID, data = dose_SUVPEAK,
    na.action = na.omit, method = "ML")
anova(fit0, fit1)
fit2 <- lm(formula = S1Peak ~ response, data = dose_SUVPEAK,
    na.action = na.omit, method = "ML")
anova(fit1, fit2)
fit3 <- lm(formula = S1Peak ~ 1, data = dose_SUVPEAK, na.action = na.omit,
    method = "ML")
AIC(fit1, fit3)
```

Summary of mixed model

The summary of the model can be reported after creating the model using the REML-method, as shown below. The coefficients are given, along with the exponents of the coefficients.

```
fitfinal.SUVPeak <- lme(fixed = S1Peak ~ response, random = ~1 |
    ID, data = dose_SUVPEAK, na.action = na.omit, method = "REML")
summary(fitfinal.SUVPeak)
i <- intervals(fitfinal.SUVPeak)$fixed
e <- exp(i)
VarCorr(fitfinal.SUVPeak)

paste0("Coefficients: ", round(i[2, 2], 2), " (", round(i[2,
    1], 2), "-", round(i[2, 3], 2), ")")
paste0("Exp: ", round(e[2, 2], 2), " (", round(e[2, 1], 2), "-",
    round(e[2, 3], 2), ")")
```

Assumptions

The assumptions must be tested (i.e. the residuals are plotted)

```
plot(fitfinal.cat.SUVPeak)
plot(fitfinal.cat.SUVPeak, ID ~ resid(.))
plot(fitfinal.cat.SUVPeak, S1Peak ~ fitted(.) | ID, abline = c(0,
    1))
plot(ranef(fitfinal.cat.SUVPeak))
```

Adding interaction with tumortype

The first model was altered to include tumortype as an interaction. Based on the AIC, this did not improve the model.

```
fit1.i.BM <- lme(fixed = S1Peak ~ response * BM, random = ~1 |
    ID, data = dose_SUVPEAK, na.action = na.omit, method = "ML")
AIC(fit1, fit1.i.BM)

summary(fit1.i.BM)
```

Output (geometric means)

As the data was first log-transformed, the average dose per group can be transformed back. However, due to the log-transformation, the values reported are geometric means.

```
print("SUVpeak")
est.temp <- lsmeans(fitfinal.cat.SUVPeak, ~response.cat)
est.temp.sum <- summary(est.temp)
est.temp.sum <- exp(est.temp.sum[, c(2, 5, 6)])
r <- apply(est.temp.sum %>%
    mutate(response.cat = levels(dose_SUVPEAK$response.cat)),
    1, function(x) {
        print(paste0(x[[4]], ": ", round(as.numeric(x[1]), 2),
            " (", round(as.numeric(x[2]), 2), "-", round(as.numeric(x[3]),
                2), ")"))
    })
```

Plots

The following two plots represent the different response categories and their SUV-peak values (plot 1) and the SUV-peak values accross response categories AND tumor type (plot 2)

```
Fig1 <- ggplot(data = dose_SUVPEAK, aes(x = response.cat, y = S1Peak.orig)) +
    theme_bw() + labs(x = "Response category", y = expression(SUV[peak] ~
    at ~ baseline)) + geom_boxplot() + scale_y_log10() + theme(panel.border = element_rect(color = NA),
    axis.line = element_line(color = "black"), legend.justification = c(0,
        0), legend.position = c(0.01, 0.01)) + ggtitle(expression(SUV[peak] ~
    per ~ response ~ category))
```

```r
cairo_ps("Fig1.eps", width = 5, height = 4)
print(Fig1, newpage = FALSE)
dev.off()

png("Fig1.png", width = 5, height = 4, units = "in", res = 300)
print(Fig1, newpage = FALSE)
dev.off()

Fig2 <- ggplot(data = dose_SUVPEAK %>%
    group_by(metastasis_type, response.cat) %>%
    summarise(z.mean = mean(S1Peak.orig), z.count = n_distinct(S1Peak.orig)),
    aes(x = metastasis_type, y = response.cat, group = 1)) +
    theme_bw() + labs(x = "Tumor type", y = "Response category",
    color = expression(Mean ~ SUV[peak])) + geom_point(aes(color = z.mean,
    size = z.count)) + scale_x_discrete(limits = c("PT", "LM",
    "BM", "VM"), labels = c("Primary", "Lymph nodes", "Bone metastases",
    "Visceral metastases")) + scale_color_gradient(low = "#edd958",
    high = "#c91212") + scale_size_continuous(name = "Tumor count",
    range = c(7, 20), guide = NULL) + geom_label(aes(label = z.count),
    alpha = 0, label.size = NA) + theme(panel.border = element_rect(color = NA),
    legend.background = element_rect(fill = NA), axis.line = element_line(color = "black"),
    legend.justification = "center", legend.position = "right",
    panel.grid = element_blank()) + ggtitle("Dose vs. Response per tumor") +
    geom_hline(yintercept = seq(0.5, 5, by = 1), color = "gray",
        size = 0.6, alpha = 0.5) + geom_vline(xintercept = seq(0.5,
    5, by = 1), color = "gray", size = 0.6, alpha = 0.5)

print(Fig2)
```
